# Wound repair and anti-inflammatory potential of *Lonicera japonica *in excision wound-induced rats

**DOI:** 10.1186/1472-6882-12-226

**Published:** 2012-11-23

**Authors:** Wei-Cheng Chen, Shorong-Shii Liou, Thing-Fong Tzeng, Shiow-Ling Lee, I-Min Liu

**Affiliations:** 1 Department of Bioengineering, Tatung University, Taipei City, Taiwan; 2 Department of Pharmacy & Graduate Institute of Pharmaceutical Technology, Tajen University, Yanpu Township, Pingtung County, Taiwan; 3 Department of Internal Medicine, Pao Chien Hospital, Ping Tung City, Pingtung County, Taiwan

## Abstract

**Background:**

*Lonicera japonica* Thunb. (Caprifoliaceae), a widely used traditional Chinese medicinal plant, is used to treat some infectious diseases and it may have uses as a healthy food and applications in cosmetics and as an ornamental groundcover. The ethanol extract of the flowering aerial parts of *L. japonica* (LJEE) was investigated for its healing efficiency in a rat excision wound model.

**Methods:**

Excision wounds were inflicted upon three groups of eight rats each. Healing was assessed by the rate of wound contraction in skin wound sites in rats treated with simple ointment base, 10% (w/w) LJEE ointment, or the reference standard drug, 0.2% (w/w) nitrofurazone ointment. The effects of LJEE on the contents of hydroxyproline and hexosamine during healing were estimated. The antimicrobial activity of LJEE against microorganisms was also assessed. The *in vivo* anti-inflammatory activity of LJEE was investigated to understand the mechanism of wound healing.

**Results:**

LJEE exhibited significant antimicrobial activity against *Staphylococcus aureus*, *Staphylococcus epidermidis*, *Escherichia coli*, *Candida albicans*, and *Candida tropicalis*. The ointment formulation prepared with 10% (w/w) LJEE exhibited potent wound healing capacity as evidenced by the wound contraction in the excision wound model. The contents of hydroxyproline and hexosamine also correlated with the observed healing pattern. These findings were supported by the histopathological characteristics of healed wound sections, as greater tissue regeneration, more fibroblasts, and angiogenesis were observed in the 10% (w/w) LJEE ointment-treated group. The results also indicated that LJEE possesses potent anti-inflammatory activity, as it enhanced the production of anti-inflammatory cytokines that suppress proinflammatory cytokine production.

**Conclusions:**

The results suggest that the antimicrobial and anti-inflammatory activities of LJEE act synergistically to accelerate wound repair.

## Background

The function of skin is to serve as a protective barrier against the environment. Wounds are physical injuries that result in an opening or break of the skin. Wound healing involves continuous cell-cell and cell-matrix interactions that allow the process to proceed in three overlapping phases: inflammation (0–3 days), cellular proliferation (3–12 days), and remodeling (3–6 months) [1]. During the inflammatory stage, neutrophils and macrophages infiltrate the wound site and phagocytose infectious agents, and the degraded tissue fragments release proteases and various reactive oxygen species (ROS) into the wound environment [2]. They also are both major sources and targets of pro-inflammatory cytokines such as tumor necrosis factor alpha (TNFα) and interleukin (IL)-1, which have been revealed as key mediators that promote necrosis factor-κB activation and ROS production during cutaneous inflammatory processes [3]. ROS play crucial roles in cell signaling and immune responses, but they also cause oxidative stress at higher levels during wound healing. Wound healing has been associated with a decrease in pro-inflammatory cytokine levels [4].

*Lonicera japonica* Thunb. (Caprifoliaceae), a widely used traditional Chinese medicinal plant that is also calledJin Yin Hua, Ren Dong, and Japanese honeysuckle, is native to eastern Asia, including Japan, Korea, northern and eastern China, and Taiwan, but it is a major invasive species in North America. *L. japonica* is traditionally used as a medicinal plant [5]. However, it is also used as a food, cosmetic and ornamental groundcover, and it can be consumed as a healthy beverage [6]. The ethnopharmacological studies and clinical findings have demonstrated that* L. japonica* possesses many biological functions, including hepatoprotective, cytoprotective, antimicrobial, antioxidative, antiviral, and anti-inflammatory properties [6]. The major parts of this plant have medicinal properties: the flower buds have anticancer and anti-inflammatory properties [7], the leaves have antioxidant and tyrosinase-inhibitory properties [8], and the stem has tyrosinase-inhibitory, xanthine oxidase-inhibitory, and nitrite-scavenging activities [9].

The potent anti-inflammatory and ethnopharmacological properties of *L. japonica* make it an excellent source of novel medicinal targets for wound healing, as there are currently no studies of the effects of such herbs on healing chronic wounds. Therefore, the present study assessed the effects of the ethnobotanical use of *L. japonica* on various parameters and stages of the wound healing process, and its antimicrobial activity was evaluated to clarify the mechanism by which *L. japonica* promotes wound healing.

## Methods

### Plant material and extraction

The flowering aerial parts of *L. japonica* were collected from Ligang Township (Pingtung County, Taiwan) in October 2010. Macroscopic and microscopic examinations, thin-layer chromatography, and high-performance liquid chromatography (HPLC) were used to confirm the authenticity of the plant material provided (this analysis was performed by Dr. Hong T.Y., Department of Biotechnology, Collage of Pharmacy and Health Care, Tajen University). The voucher specimen (Lot No. LJ 20101021) has been preserved in our laboratory for future reference.

The flowering aerial parts of *L. japonica* were air-dried, pulverized to a coarse powder in a mechanical grinder, and passed through a 40-mesh sieve to get powdered samples. Powdered samples (5 kg) were extracted at room temperature thrice with 10 L of 95% ethanol for 48 h on an orbital shaker to make the ethanol extracts. The *L. japonica* ethanol extract (LJEE) was evaporated to dryness under reduced pressure to completely eliminate alcohol and lyophilized, yielding approximately 563 g of dry residue (w/w yield: 11.3%). LJEE was stored at −20°C until use and suspended in distilled water. The chlorogenic acid content of the samples was then analyzed using an HPLC protocol, and the antimicrobial activity was screened as described in subsequent sections.

### Chlorogenic acid content analysis

The chlorogenic acid content in was quantified using an Agilent 1200 Series HPLC system with a Thermo Scientific BDS Hypersil Phenyl Column (4.6 mm × 100 mm). The mobile phase of the optimized chromatographic method consisted of solvent A (methanol) and solvent B (0.5% (v/v) acetic acid in water). The elution profile was as follows: 0 min 10% A in B, 28.6 min 60% A in B, 30 min 10% A in B. The mobile phase was passed under vacuum through a 0.45-μm membrane filter before use. The flow rate was 1 ml/min, and injection volume was 10 mL. Absorption was measured at 280 nm. The identity of chlorogenic acid was determined by matching the UV spectrum and retention time with those of the standard. Chlorogenic acid (purity ≥ 95.0%, Sigma-Aldrich, Inc., St. Louis, MO, USA; Cat. No. C3878) was used at the concentrations of 12.5 to 400 μg/ml to construct the calibration curve. The retention time of this compound was 12.4 min. The amount of chlorogenic acid present was quantified using the peak areas. Chlorogenic acid content was expressed as μg/g of the dry extract. All samples were performed in triplicate.

### Antimicrobial activity

The agar diffusion method was used to evaluate the antimicrobial activity [10]. The microorganisms used for the antimicrobial activity were *Staphylococcus aureus* (ATCC 29213), *Staphylococcus epidermidis* (ATCC 155), *Escherichia coli* (ATCC 23815), *Candida albicans* (ATCC 90028), and *Candida tropicalis* (ATCC 20401). These organisms were identified and procured from the Bioresource Collection and Research Center of the Food Industry Research and Development Institute (Hsinchu, Taiwan).

Bacteria were cultured overnight at 37°C in Mueller Hinton Broth (Oxoid), and fungi were cultured at 28°C for 72 h in Potato Dextrose Broth (Oxoid). The final inoculum size was 100 μl, and the inocula consisting of suspensions containing 1×10^8^ CFU/ml bacteria or 1×10^4^ spore/ml fungi were spread onto Mueller Hinton Agar and Potato Dextrose Agar medium, respectively. Each disc (6 mm in diameter) was impregnated with 10 μl of 100 mg/ml (1 mg/disc) LJEE. Gentamicin (10 μg/disc) and tetracycline (10 μg/disc) were used as positive controls for bacteria, and fluconazole (10 μg/disc) and ketoconazole (10 μg/disc) were used as positive controls for fungi. Gentamicin (Cat. No. G1914), tetracycline (Cat. No. T3258), fluconazole (Cat. No. F8929), and ketoconazole (Cat. No. K1003) were obtained from Sigma-Aldrich, Inc. The test plates were incubated at 37°C for 24 h for bacteria and at 28°C for 72 h for fungi depending on the incubation time required for visible growth. The minimum inhibitory concentration (MIC) values were also studied for microorganisms that exhibited sensitivity to LJEE in the disc diffusion assay. Sterile filter paper discs (6 mm in diameter) containing 2.5-1000 μg/disc of all the components were placed on the surface of the medium. The MIC was defined as the lowest concentration of extract that inhibited visible growth on agar.

### Experimental animals

Male Wistar rats (200–250 g, 6–8 weeks old) were obtained from the National Laboratory Animal Center (Taipei, Taiwan). They were maintained in a temperature-controlled room (25 ± 1°C) and kept on a 12:12 light–dark cycle in our animal center. Food and water were available *ad libitum*. The rats were used after acclimatization to the laboratory environment for a 7-day period. All animal procedures were performed according to the Guide for the Care and Use of Laboratory Animals of the National Institutes of Health as well as the guidelines of the Animal Welfare Act. These studies were conducted with the approval of the Institutional Animal Care and Use Committee (IACUC) at Tajen University (approval number: IACUC 99–24; approval date: December 23, 2010).

### Excision wound model

Three groups with eight animals in each group were anaesthetized by the open mask method with anesthetic ether. The backs of the rats were depilated. One excision wound was inflicted by cutting away a 500-mm^2^ section of the full thickness of the skin from a predetermined area. The wound was left undressed to the open environment. Group 1 rats were dressed with simple ointment composed of 5% (w/w) wool fat, 5% (w/w) hard paraffin, 5% (w/w) cetostearyl alcohol, and 85% (w/w) white soft paraffin. Wounds of experimental animals (Group 2 and 3) were treated with the 10% (w/w) LJEE ointment and Nitrofurazone ointment (0.2%, w/w), respectively. The 10% (w/w) LJEE ointment was composed of 10 g of LJEE incorporated into 100 g of a simple ointment base. Nitrofurazone ointment (0.2%, w/w) (GSK Pharmaceuticals, Bangalore, India) was used as a reference standard drug to assess the wound-healing potential of the LJEE ointment. Simple ointment, LJEE ointment (10%, w/w) and the reference standard drug were applied topically (dose, approximately 0.20 g/wound) once daily. Special care was taken to avoid variation in the dose given. The wound area was traced on a sheet of sterile autoclaved transparent paper (three times to get an average area) and then placed on graph paper to determine the area [11]. Wound contraction was calculated as the percentage reduction of the initial wound area. Wound healing was monitored by taking photographs on days 1, 3, 9, 12, and 15 after wounding. Wounds were considered closed (completely healed) if moist granulation tissue was no longer apparent and the wound was covered with new epithelium.

### Estimation of proinflammatory and anti-inflammatory cytokine induction

Blood samples were collected from all animals of each group on days 1 and 9 after wounding. The levels of proinflammatory (TNFα and IL-6) and anti-inflammatory cytokines (IL-10) were estimated by performing enzyme-linked immunosorbent assays (ELISAs) using commercial kits. ELISA kits for the determination of TNFα (Cat. No. ab46070), IL-6 (Cat. No. ab100772), and IL-10 (Cat. No. ab100764) were obtained from Abcam Inc. (Cambridge, MA, USA). Assays were performed according to the manufacturer’s instructions. The cytokine concentrations were determined in pg/ml by plotting the graph for the standard. All experiments were performed in triplicate to ensure the accuracy of the observations.

### Estimation of hydroxyproline and hexosamine

On days 3, 9, and 15 after wounding, a piece of skin from the healed wound area was collected and analyzed for its levels of hydroxyproline, which is the basic constituent of collagen. Tissues were dried in a hot air oven at 60-70°C to an equal weight and hydrolyzed in 6 N HCl at 130°C for 4 h in sealed tubes. The hydrolysate was neutralized to pH 7.0 with 0.1N KOH and subjected to chloramine-T oxidation for 20 min. The reaction was terminated by the addition of 0.4 M perchloric acid, and color was developed with the help of Ehrlich reagent at 60°C and measured at 557 nm using a UV/Vis spectrophotometer (Shimadzu) [12]. To estimate the hexosamine levels, the weighed granulation tissues were hydrolyzed in 6 N HCl for 8 h at 98°C, neutralized to pH 7 with 4 N NaOH, and diluted with Milli-Q water. The hexosamine content of granulation tissues was estimated as described previously with minor modifications [13]. The diluted solution was mixed with acetylacetone solution and heated to 96°C for 40 min. The mixture was cooled, and 96% ethanol was added, followed by the addition of *r*-dimethylamino-benzaldehyde solution (Ehrlich’s reagent). The solution was thoroughly mixed and kept at room temperature for 1 h, and the absorbance was measured at 530 nm using a double-beam UV/Vis spectrophotometer (Shimadzu). The amount of hexosamine was determined via comparisons with a standard curve.

### Histopathological studies

A specimen sample was isolated from each group of rats on day 15 after wounding for histopathological examination. Skin specimens were immediately fixed in 10% (v/v) neutral-buffered formalin, and the fixative solution replaced every 2 days until the tissues hardened. Each specimen was embedded in a paraffin block, and thin sections (3 μm) were prepared and stained with Masson’s trichrome (to detect collagen fibers) and hematoxylin and eosin (H&E) (for general morphological observations). Slides were examined qualitatively under a light microscope for collagen formation, fibroblast proliferation, angiogenesis, epithelization, and granulation tissue formation by employing a light to intense scale (+ to +++) [14].

### Statistical analysis

Data are expressed as the mean ± SD for each group of animals. Statistical analysis was performed with one-way analysis of variance. Dunnett’s post hoc test was used to determine the source of significant differences where appropriate. Data were analyzed using SigmaPlot (Version 11.0). A P value < 0.05 was considered statistically significant.

## Results

### Quantitative analysis

**Figure 1:**
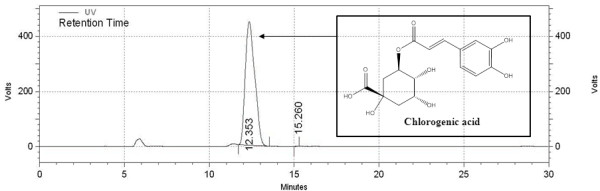
HPLC chromatogram of LJEE (280 nm).

The calibration equation of peak area against the concentration of chlorogenic acid was y = 7286.7x+124.6 (*R*^2^ = 0.9998). The chromatogram of the sample solution is shown in Figure 1. The chlorogenic acid content in LJEE was 64.2 ± 0.18 μg/g of the dry extract.

### Antimicrobial activity

LJEE inhibited the growth of all organisms tested, but the efficiency of its inhibition was organism-specific. The zone of inhibition of LJEE ranged from 8.17 to 12.87 mm. *Candida tropicalis* (12.87 ± 0.09 mm) was the most sensitive to LJEE, followed by *Staphylococcus aureus* (10.96 ± 0.08 mm), *Staphylococcus epidermidis* (10.67 ± 0.11 mm), *Candida albicans* (9.32 ± 0.14 mm), and *Escherichia coli* (8.17 ± 0.09 mm). The MICs of LJEE ranged from 100 to 300 μg/disc, with the lowest MIC observed for *Candida tropicalis*.

### Effect of LJEE on wound contraction

**Figure 2:**
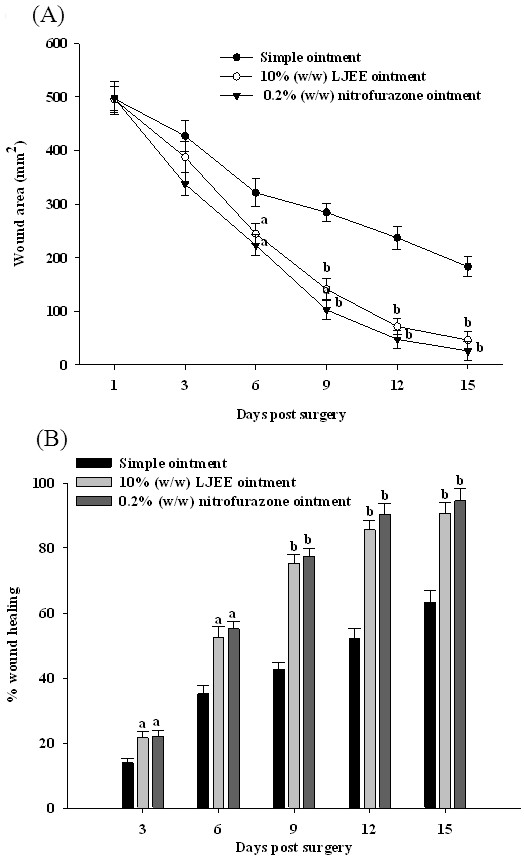
**Effect of LJEE ointment on the wound area (A) and the percentage of wound contraction (B) in excision wound models on different days after wounding. **Values (mean ± SD) were obtained for each group of eight rats. ^a^P < 0.05 and ^b^P < 0.01 compared to the values of simple ointment-treated rats on the indicated day in each group.

**Figure 3:**
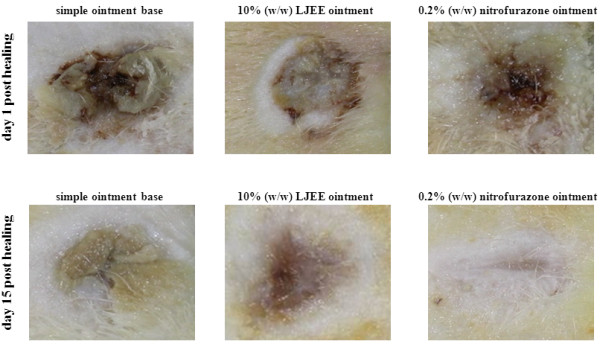
Macroscopic changes in skin wound sites in rats treated with simple ointment base, 10% (w/w) LJEE ointment, or 0.2% (w/w) nitrofurazone ointment on days 1 and 15 after wounding.

The reduction of the wound area in the different groups over a period of 15 days is presented in Figure 2A. The contraction of the wound area in the 10% (w/w) LJEE ointment-treated group, as measured on every other day, significantly increased from 21.6% on day 3 to 65.3 and 85.6% on days 9, and 12, and the wound was completely healed on day 15. The effects were similar in rats that received 0.2% (w/w) nitrofurazone ointment treatment (Figure 2B). In comparison, the simple ointment-treated group exhibited 13.9, 42.7, 52.2, and 63.2% contraction on days 3, 9, 12, and 15, respectively (Figure 2B). The macroscopic changes of the site of the wound between days 1 and 15 are shown in Figure 3.

### Effect of LJEE on proinflammatory cytokines (TNFα and IL-6) production

After wounding, the TNFα level in the simple ointment-treated group (day 1: 243.7 ± 18.8 pg/ml; day 9: 216.4 ± 22.5 pg/ml) was significantly higher than that in the 0.2% (w/w) nitrofurazone ointment-treated group (day 1: 203.5 ± 20.9; day 9: 143.7 ± 21.3 pg/ml; Table 1). The TNFα level on day 1 after wounding in the LJEE ointment-treated group was 227.8 ± 19.4 pg/ml, which was not different from that in the simple ointment-treated group (Table 1). On day 9 after wounding, the TNFα level in the LJEE ointment-treated group (155.3 ± 17.9 pg/ml) was significantly (P < 0.01) lower than that in the group treated with simple ointment (Table 1).

**Table 1:** Effects of LJEE ointment on the production of TNFα, IL-6, and IL-10 in healed wounds on days 1 (d1) and 9 (d9) after wounding

**Treatments**	**Post-wounding day**	**TNFα (pg/ml)**	**IL-6 (pg/ml)**	**IL-10 (pg/ml)**
Simple ointment	d1	243.7 ± 18.8	104.2 ± 16.4	432.7 ± 25.8
	d9	216.4 ± 22.5	93.5 ± 15.8	547.9 ± 31.4
10% (w/w) LJEE ointment	d1	227.8 ± 19.4	88.1 ± 12.3	784.0 ± 28.3^a^
	d9	155.3 ± 17.9^b^	79.2 ± 17.3^a^	1038.6 ± 49.7^b^
0.2% (w/w) nitrofurazone ointment	d1	203.5 ± 20.9^a^	67.0 ± 14.9^b^	811.5 ± 30.7^b^
	d9	143.7 ± 21.3^b^	40.6 ± 13.7^b^	1235.7 ± 41.2^b^

Values (mean ± SD) were obtained from each group of 8 animals. ^a^ P < 0.05 and ^b ^P < 0.01 compared to the values of simple ointment-treated rats on the indicated day in each group, respectively.

At 24 h after wounding, the IL-6 level in LJEE ointment-treated rats (88.1 ± 12.3 pg/ml) was slightly lower than that in the simple ointment-treated group (104.2 ± 16.4 pg/ml), whereas the IL-6 levels was significantly reduced (P < 0.05) to 79.2 ± 17.3 pg/ml on day 9 after wounding in the LJEE ointment-treated group (Table 1). Conversely, a high IL-6 level (93.5 ± 15.8 pg/ml) was observed on day 9 after wounding in simple ointment-treated rats. The IL-6 level in the 0.2% (w/w) nitrofurazone ointment-treated group (67.0 ± 14.9 pg/ml) on day 1 after wounding was significantly (P < 0.01) lower than that in the simple ointment-treated group, and the reduction in IL-6 levels was sustained (40.6 ± 13.7 pg/ml) on day 9 after wounding (Table 1).

### Effect of LJEE on anti-inflammatory cytokine (IL-10) production

The IL-10 level after wounding in the simple ointment-treated group (day 1: 432.7 ± 25.8 pg/ml; day 9: 547.9 ± 31.4 pg/ml) was significantly (P < 0.01) lower than that in the 0.2% (w/w) nitrofurazone ointment-treated group (day 1: 811.5 ± 30.7 pg/ml; day 9: 1235.7 ± 41.2 pg/ml; Table 1). The IL-10 level in the 10% (w/w) LJEE ointment treated group was markedly elevated to 784.0 ± 28.3 and 1038.6 ± 49.7 pg/ml on days 1 and 9 after wounding, respectively (Table 1).

### Effect of LJEE on the hydroxyproline and hexosamine contents during healing

The hydroxyproline and hexosamine contents in healing wounds on different days after wounding are presented in Table 2. The hexosamine content was significantly higher on days 3 and 9 after wounding in the LJEE and nitrofurazone ointment-treated groups than in the simple ointment-treatment group. On days 15 after wounding, the hexosamine content in the LJEE and nitrofurazone ointment-treated groups was still higher than that in the simple ointment-treatment group. Similarly, hydroxyproline content was significantly higher in the LJEE and nitrofurazone ointment-treated groups than in the simple ointment-treated group throughout the course of healing (Table 2).

**Table 2:** Effects of LJEE ointment on the hexosamine and hydroxyproline content in granulation tissue on different days after wounding

**Treatments**	**Hexosamine (mg/100 mg of tissue)**			**Hydroxyproline (mg/g tissue)**		
	**3rd day**	**9th day**	**15th day**	**3rd day**	**9th day**	**15th day**
Simple ointment	0.20 ± 0.04	0.47 ± 0.06	0.68 ± 0.09	21.57 ± 1.17	33.51 ± 1.83	44.8 ± 2.17
10% (w/w) LJEE ointment	0.36 ± 0.06^a^	0.69 ± 0.08^a^	0.79 ± 0.07^a^	38.32 ± 2.03^b^	50.23 ± 2.11^b^	71.8 ± 2.46^b^
0.2% (w/w) nitrofurazone ointment	0.41 ± 0.09^b^	0.76 ± 0.05^b^	0.85 ± 0.06^a^	41.46 ± 1.92^b^	60.64 ± 2.42^b^	83.4 ± 2.73^b^

Values (mean ± SD) were obtained from each group of 8 animals. ^a^ P < 0.05 and ^b ^P < 0.01 compared to the values of simple ointment-treated rats in each group, respectively.

### Effect of LJEE on the histopathological features of healed wounds

**Figure 4:**
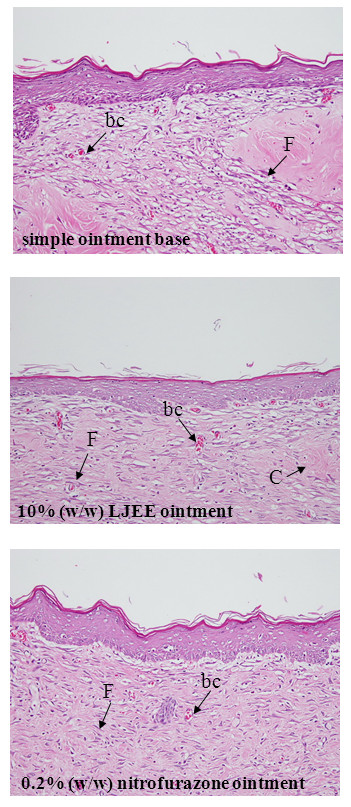
**Histological examination of healed wound sections stained with H&E. The photomicrographs show healed wound sections isolated from rats treated with simple ointment base, 10% (w/w) LJEE ointment, or 0.2% (w/w) nitrofurazone ointment on day 15 after wounding. **The photomicrographs were taken at a magnification of ×200. Abbreviation: bc, blood capillaries; C, collagen fibers; F, fibroblast. The histopathological scores of the healed wounds are presented in Table 3.

The histopathological characteristics of the healed wounds on day 15 after wounding are shown in Figure 4. Greater tissue regeneration was observed in the nitrofurazone ointment-treated group, as demonstrated by the complete epithelization, significantly higher collagen deposition, and presence of granulation tissues. Conversely, the tissue obtained from the control group that received simple ointment exhibited disorganized fibroblasts, less collagen fiber deposition, and angiogenesis. The wounds treated with LJEE ointment exhibited less scar formation, enhanced fibroblast proliferation, newly formed blood capillaries (angiogenesis), and re-epithelialization. The scoring of the characteristic histopathological features of the healed wounds of the experimental animals is presented in Table 3.

**Table 3:** The histopathological scores on healed wounds from LJEE and nitrofurazone ointment-treated animals

**Treatments**	**Collagen formation**	**Fibroblast proliferation**	**Angiogenesis**	**Granulation tissue**	**Re-epithelization**
Simple ointment	+	+	++	+	+
10% (w/w) LJEE ointment	+++	+++	++	+++	+++
0.2% (w/w) nitrofurazone ointment	+++	+++	+++	+++	+++

Values were obtained from each group of 8 animals.

## Discussion

The significant reduction in wound size after LJEE ointment treatment was correlated with the histopathological findings of increased epithelization activity, angiogenesis, granulation tissue formation, and finally remodeling of the extracellular matrix. Collagen not only confers strength and integrity to the tissue matrix but also plays an important role in homeostasis and epithelialization in the later stages of wound healing [15]. Hydroxyproline is an uncommon amino acid present in the collagen fibers of granulation tissues. Biochemical analysis revealed increased hydroxyproline content, which is a reflection of increased cellular proliferation and therefore increased collagen synthesis, after LJEE ointment treatment [16]. Increased hexosamine content reflects the stabilization of collagen molecules via enhanced electrostatic and ionic interactions [16]. Hence, enhanced hydroxyproline and hexosamine synthesis provides strength to repaired tissue and stimulates healing. Significant increases in hydroxyproline and hexosamine content were observed in the wounds after LJEE ointment, and these findings were supported by the histopathological data. The potent wound-healing capacity of LJEE, as evidenced by the wound contraction and increased levels of biochemical parameters in healing tissue, has thus validated the ethnotherapeutic claim.

Open wounds are particularly prone to infection, especially by bacteria, and they also provide an entry point for organics that cause systemic infections. Infected wounds heal less rapidly, and infection often results in the formation of unpleasant exudates and the production of toxins concomitantly with the killing of regenerating cells [17]. Wide ranges of antibiotics are presently being used to treat wound infections in humans [18]. However, due to their adverse effects and the presence of antibiotic-resistant organisms, researchers are now investigating the extracts of biologically active compounds isolated from plant species that are used in herbal medicine [19]. The use of LJEE to treat various skin infections is justified by this work, as LJEE exhibited commendable activity against all the organisms tested. The external application of LJEE on wounds prevented the invasion of microbes through the wound, resulting in protection of the wound against infection by various organisms.

Strong TNFα and IL-6 induction after cutaneous injury was observed as early as 12–24 h after wounding, and these components play a major role in the inflammatory phase of wound healing by enhancing angiogenesis [20]. Our study revealed that TNFα and IL-6 levels were slightly lower at 24 h after wounding in LJEE ointment-treated animals. It is thus apparent that the LJEE ointment did not interfere with macrophage-derived proinflammatory cytokines during the first stage of healing. However, LJEE ointment treatment elevated IL-10 levels on days 1 and 9 after wounding. IL-10 is an anti-inflammatory cytokine produced by various cells including macrophages and T lymphocytes [21]. IL-10 appears to influence the wound-healing environment by decreasing the expression of proinflammatory/profibrotic mediators, resulting in decreased recruitment of inflammatory cells to the wound [21]. Treatment with LJEE ointment increased the serum IL-10 concentration and simultaneously downregulated TNFα and IL-6 expression, especially on day 9 after wounding. There are reports that TNFα inhibits collagen formation and hydroxyproline production, which are essential for the final part of the proliferative phase in wound healing [22]. The findings suggested that LJEE regulates anti-inflammatory and proinflammatory cytokines and ultimately the systemic immune pathways associated with them, thus leading to cellular proliferation.

In the current Chinese Pharmacopoeia, chlorogenic acid is officially used as the indicator compound to characterize the quality of this herb [23]. Considerable chlorogenic acid content has been detected in LJEE. It has been demonstrated that chlorogenic acid strongly inhibits the production of TNFα and IL-6 by human peripheral blood mononuclear cells stimulated with staphylococcal exotoxins [24]. Chlorogenic acid inhibits the synthesis of other mediators such as IL-1, interferon-gamma, monocyte chemotactic protein-1, and macrophage inflammatory protein-1a [24]. Additionally, chlorogenic acid has strong bacteriostatic activity [25]. Hence, the synergistic effect of the antimicrobial and anti-inflammatory activities of LJEE accelerated the wound healing process.

Recent studies with other plant extracts revealed that phytochemical constituents such as flavonoids, triterpenoids, and tannins can promote the wound-healing process [26-28]. As *L. japonica* is being used and cultivated in more countries, its chemical components have been extensively studied. Essential oil, organic acids, flavones, saponins, iridoids, and inorganic elements were isolated and identified as the primary components [5]. Among them, essential oil and chlorogenic acid have demonstrated pharmacological effects, and they are considered the active compounds of *L. japonica*[5].

## Conclusions

The results revealed the potential use of LJEE as an external treatment for wounds. The mechanism of action of LJEE was postulated to involve angiogenesis, collagen deposition, granulation tissue formation, epithelization, and wound contraction at the proliferative stage, and these actions are attributed to the synergistic effects of the strong antibacterial and anti-inflammatory effects of the active compounds in the extract such as chlorogenic acid. However, further study is needed to isolate the active ingredients that promote wound healing before LJEE can be used clinically. As *L. japonica* is ubiquitously and abundantly grown, it could be a fairly economical therapeutic agent for wound management as a prohealer as well as a modality for controlling abnormal healing.

## Competing interests

The authors declare that they have no competing interests.

## Authors’ contributions

WCC carried out the experimentation as part of PhD study. SSL contributed to study design, data interpretation and manuscript writing. TTF performed the experiments and analysis and participated to data interpretation. SLL supervised the work and evaluated the data. IML supervised the work, evaluated the data, manuscript writing and corrected the manuscript for publication. All authors read and approved the final manuscript.

## Pre-publication history

The pre-publication history for this paper can be accessed here:

http://www.biomedcentral.com/1472-6882/12/226/prepub
